# Diagnosis and treatment of infective endocarditis in pregnancy: a case report

**DOI:** 10.1186/s13019-020-01147-6

**Published:** 2020-05-24

**Authors:** Jing Wang, Anlong Wang, Yong Cui, Ceng Wang, Jian Zhang

**Affiliations:** 1 Department of Ultrasound, Zhejiang Provincial People’s Hospital, People’s Hospital of HangZhou Medical College, Hangzhou, 310014 Zhejiang China; 2 Department of Radiology, Zhejiang Provincial Integrated Chinese and Western Medicine Hospital, Hangzhou Red Cross Hospital, Hangzhou, China; 3 Department of Cardiothoracic Surgery, Zhejiang Provincial People’s Hospital, People’s Hospital of HangZhou Medical College, Hangzhou, China

**Keywords:** Pregnancy complications, cardiovascular, Endocarditis, bacterial, Embolism, Heart valve diseases, Echocardiography, Surgery

## Abstract

**Background:**

Pregnancy with infective endocarditis (IE) is rare, but the fetal and maternal mortality rates of these pregnancies are very high, making IE a serious threat to the safety of pregnant women and their fetuses. Therefore, for pregnant women with recurrent fever, a detailed medical history and physical examination should be performed, echocardiography and blood culture should be carried out as soon as possible, multidisciplinary consultation should be implemented, and a diagnosis and treatment plan should be formulated right away, as this is key to saving the lives of mothers and infants.

**Case introduction:**

A 30-year-old pregnant Chinese woman had IE at 26 weeks of gestation. After close monitoring and care until 31 weeks of gestation, she underwent a successful delivery, cardiac surgery, repair of the patent ductus arteriosus (PDA), mitral valvuloplasty (MVP) and removal of the vegetations. The operation was successful, and further follow-up evaluation showed no abnormality.

**Conclusion:**

For the diagnosis and treatment of IE in pregnancy, it is of great importance to implement an individualized diagnosis and treatment plan in combination with close monitoring by echocardiography and to select the right time for cardiac surgery and termination of pregnancy.

## Background

Pregnancy complicated by IE is rare, the condition is dangerous, and its clinical symptoms and signs lack specificity. Because of the increased blood volume and hemodynamic changes that characterize pregnancy, the endocardial vegetation is impacted by high-pressure blood flow, which easily drops and leads to systemic or pulmonary circulation embolism. Pregnant women may have respiratory or cardiac arrest, sudden death, or abscesses in various organs, among other problems; additionally, the fetus may experience intrauterine distress, and a stillbirth can occur at any time. At present, there are many questions regarding the diagnosis and treatment of IE in pregnancy. We report a woman with a pregnancy complicated by IE at 26 weeks of gestation who was closely monitored via a multidisciplinary collaboration. After delivery at 31 weeks of gestation, heart surgery was successfully performed.

## Case report

A 30-year-old Chinese woman was hospitalized at 26 weeks after her last period and had experienced repeated fever for 2 months. The fevers occurred without inducement, her maximum body temperature was 39.3 °C, and her fevers were accompanied by coughing, expectoration and occasional vomiting. Therefore, she was examined in the local hospital. Because the cause of the fever was unknown after the initial investigation, she was given an intravenous drip of amoxicillin and clavulanate potassium. After 1 week of hospitalization, her body temperature slightly decreased, so she took amoxicillin orally. Over the previous 2 months, the patient has been repeatedly feverish, coughing and expectorating. After echocardiography suggested infective endocarditis (IE), it was recommended that she be transferred to a more appropriate hospital for further treatment. Thus, the patient was transferred to our hospital for treatment.

Her body temperature was 36.5 °C, her heart rate was 86 bpm, and her blood pressure was 116/62 mmHg. The fetal heart rate was 146 bpm, and fetal movement was evident. A continuous loud noise was heard through the left edge of the patient’s sternum.

**Fig. 1:**
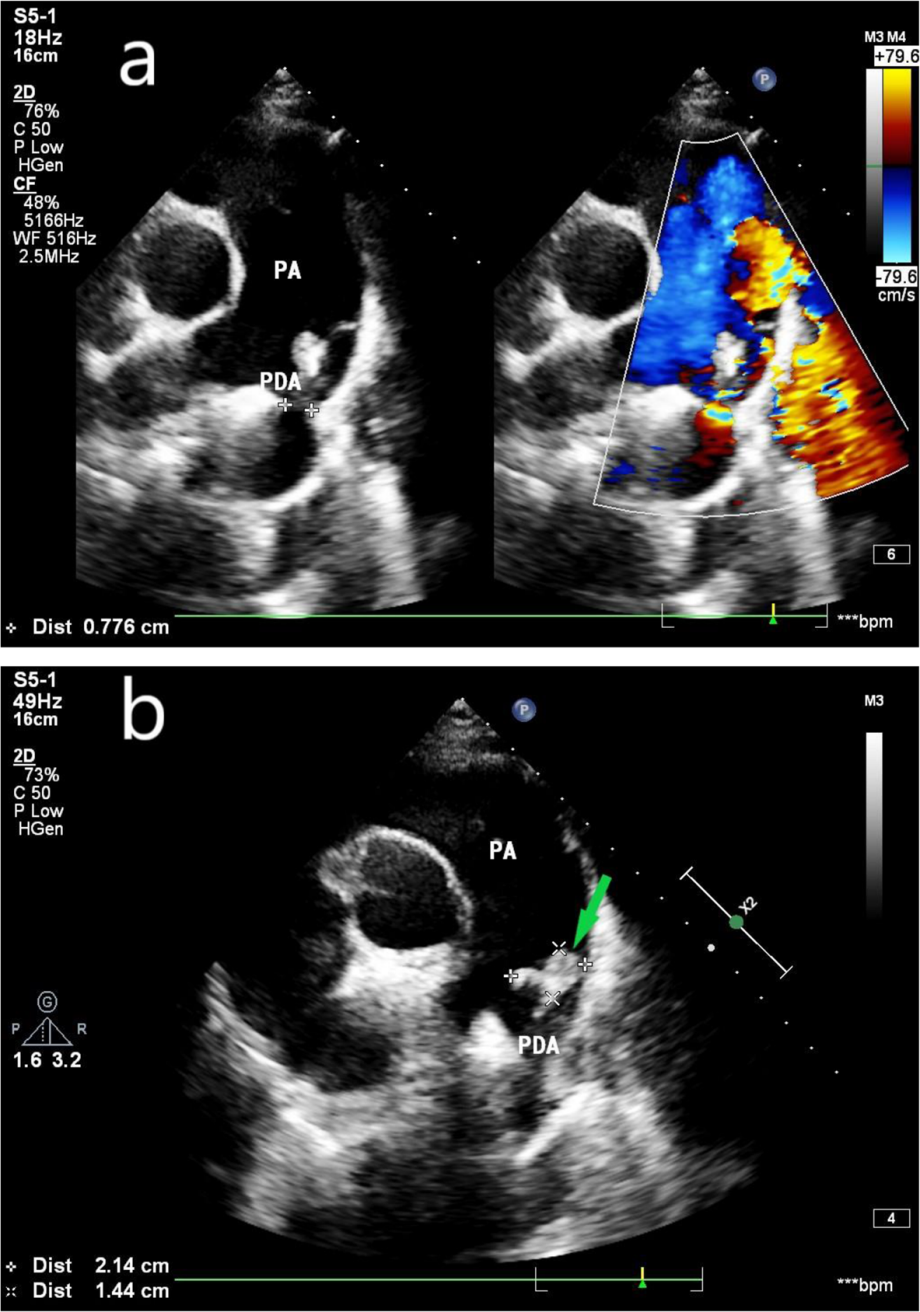
**a** Echocardiography at 26 weeks of gestation shows patent ductus arteriosus between the descending aorta and left pulmonary artery; **b** at the opening of the PDA, there is a vegetation (PA = pulmonary artery; PDA = patent ductus arteriosus; white arrow = infective endocarditis vegetative)

**Fig. 2:**
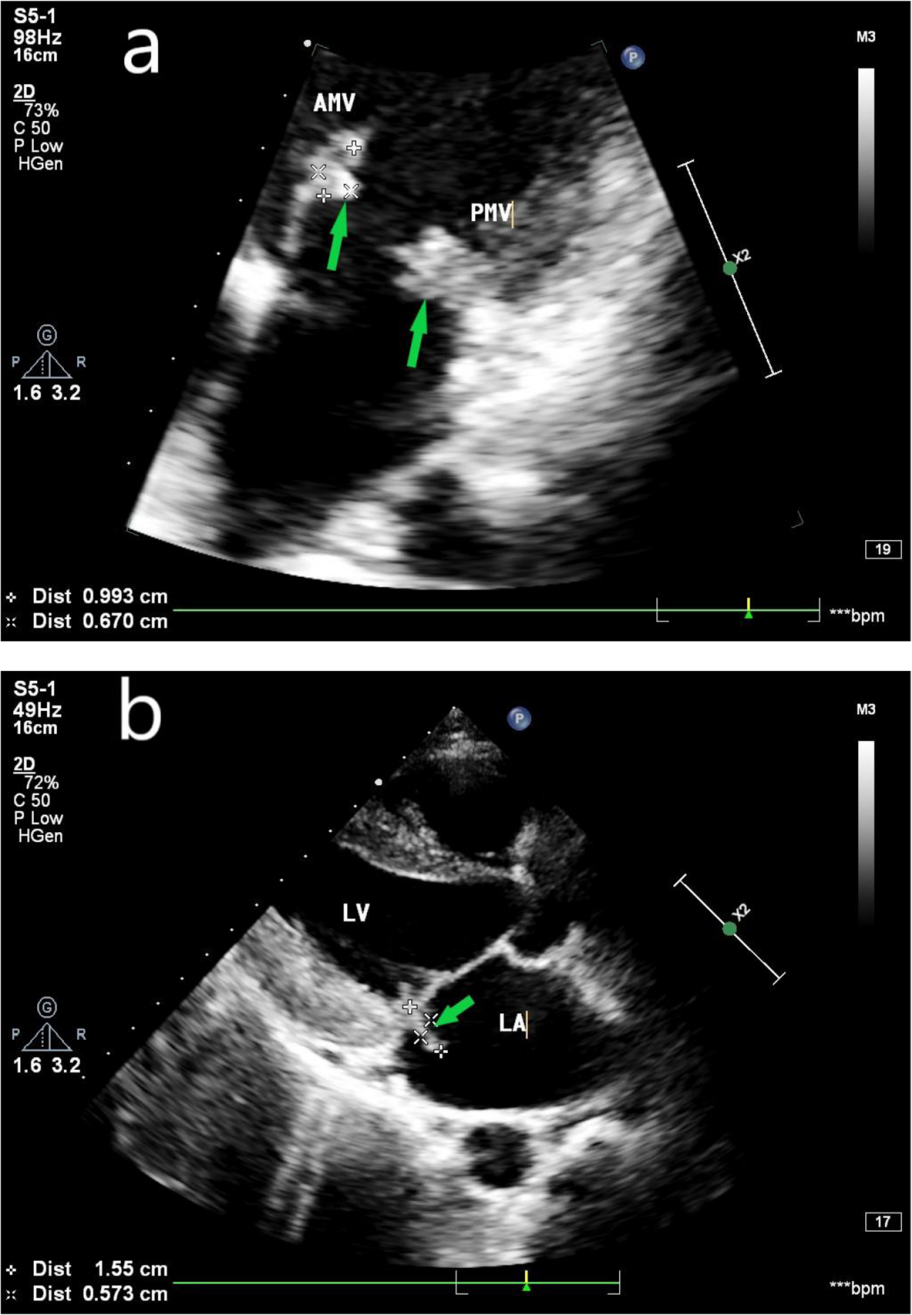
**a** Echocardiography at 26 weeks of gestation shows that the anterior leaflet of the mitral valve is thickened, is rough, and has vegetative formation. **b**. The posterior leaflet shows vegetative formation (AMV = anterior leaflet of the mitral valve; PMV = posterior leaflet of the mitral valve; LA = left atrium; LV = left ventricle; green arrow = infective endocarditis vegetative)

Echocardiography showed the following: 1. Congenital heart disease, namely, patent ductus arteriosus (PDA) and a horizontal left to right shunt of the artery, with a hyperechoic mass at the beginning of the left pulmonary artery (21 mm * 15 mm) (Fig. 1a-b), indicating the formation of IE vegetation; 2. Moderate mitral regurgitation, hyperechoic masses were attached to the mitral valve (16 mm * 6 mm, posterior leaflet; 10 mm*7 mm, anterior leaflet) (Fig. 2a-b), indicating the formation of IE vegetation; and 3. Left heart and pulmonary artery enlargement. Gram-positive Group G Streptococcus was found in the blood culture. Because blood culture and echocardiography indicated IE, we had many multidisciplinary discussions, closely observed the patient and actively treated the patient, and the patient’s condition is still stable.

**Fig. 3:**
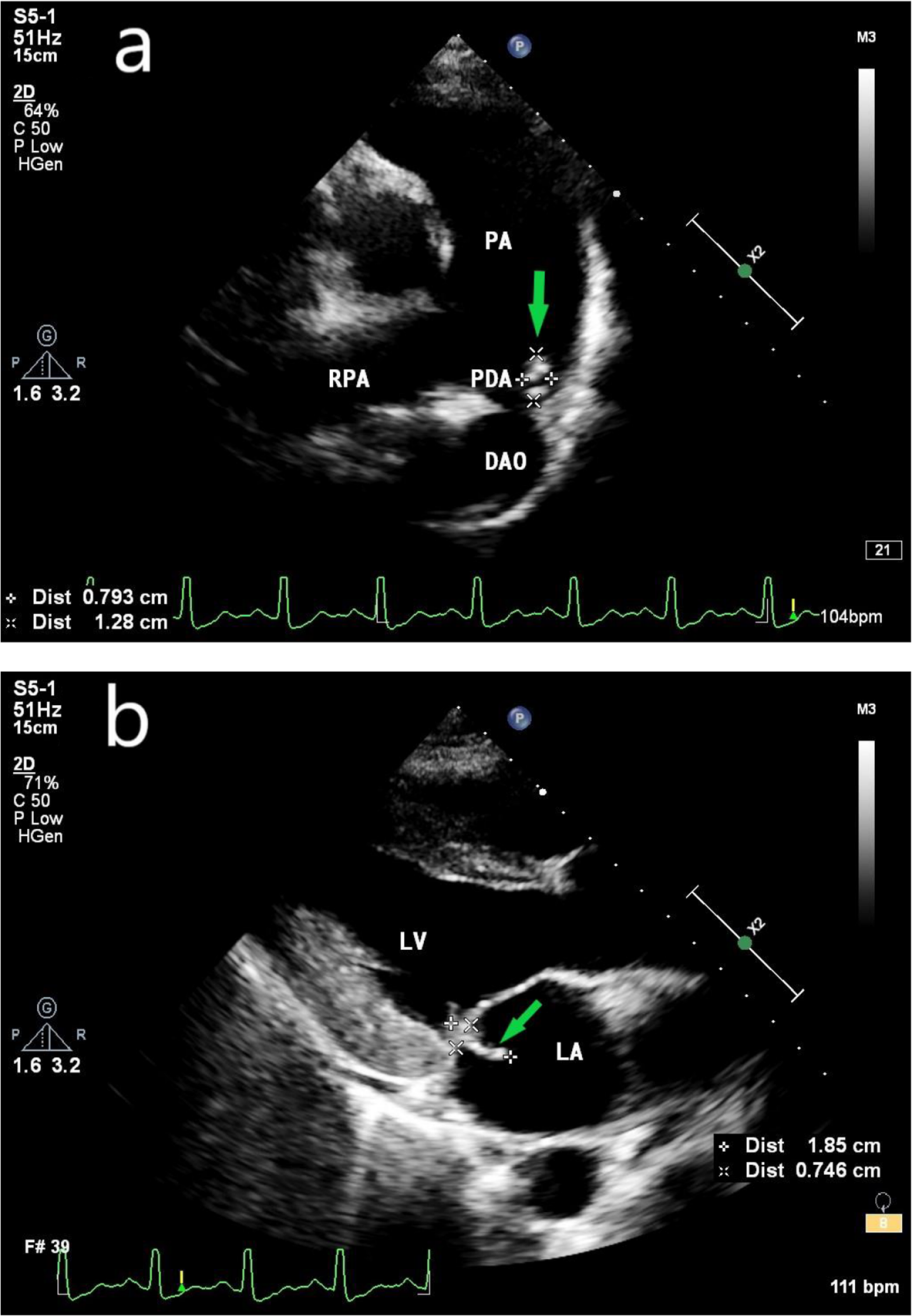
**a** Echocardiography at 31 weeks of gestation shows that the vegetation has shrunk. **b** No obvious reduction of mitral valve vegetation. (PA = pulmonary artery; PDA = patent ductus arteriosus; RPA = right pulmonary artery; DAO = descending aorta; green arrow = infective endocarditis vegetative)

At 31 weeks of gestation, the patient suddenly had chest tightness, a temperature of 37.6 °C, nausea, headache, chest pain with breathing, and a cough with white foam sputum. She vomited 1 time and had concomitant back pain. MRI examination showed that there was no obvious abnormality in the brain. Echocardiography showed that the hyperechoic mass at the beginning of the left pulmonary artery was significantly smaller than that at the previous time (13 mm * 8 mm) (Fig. 3a) and that the mass swung with the cardiac cycle. There is no obvious reduction of the mitral valve vegetation (18 mm * 7 mm, posterior leaflet; 11 mm*7 mm, anterior leaflet) (Fig. 3b). Furthermore, the blood picture indexes, except for procalcitonin, were significantly increased. If we were to consider a monistic explanation wherein a small bacterial embolus was shed, a cerebral infarction could not be completely ruled out, even though it was not indicated by the MRI. Thus, an emergency cesarean section was performed, and a live male baby was delivered in the cephalic position, with a weight of 2160 g, a length of 43 cm, and an Apgar score of 10 points.

After the operation, oxytocin was given to prevent uterine bleeding, low-molecular-weight heparin was given as an anticoagulant, and vancomycin was used an as anti-infection agent. The patient’s breathing, circulation, mental state and limb muscle strength were closely monitored, and the staff remained alert to the occurrence of pulmonary embolism or cerebral embolism. The patient’s overall condition was stable. After moderate recovery (on the 10th day after the operation), the patient underwent cardiac surgery by conventional cardiopulmonary bypass (CPB).

Cardiac surgery was performed with the repair of the PDA patch and mitral valvuloplasty. Intraoperative findings were as follows: an obviously enlarged heart and a palpable tremor on the bifurcation surface of the pulmonary artery. After heparinization, CPB was established routinely, the right atrium was cut, the ascending aorta was blocked, and the pulmonary artery trunk was cut longitudinally. The PDA was located at the beginning of the left pulmonary artery, with a diameter of approximately 0.8 cm. After the nearby vegetation was removed, an 18F catheter was inserted into the descending aorta through the PDA, 3 ml normal saline was injected into the catheter balloon, and the catheter was pulled out properly to avoid the right cardiac shunt of blood through the PDA. After the heart stopped beating satisfactorily, cool down continued to 25 °C. Through the atrial septal incision into the left atrium, the contrazygote margin of areas A3 and P3 of the mitral valve and the vegetation on the surface of the valve leaf were found. After washing was performed with plenty of normal saline repeatedly, wiping with concentrated antibiotic gauze was performed repeatedly to maintain the whole leaflet tissue. Posterior mitral valvuloplasty was performed. The mitral valve opened and closed well in the water injection test. Continuous suture of the atrial septum and right atrial incision was performed. The diameter of the bovine pericardial patch was approximately 1 cm, and the opening of the PDA was repaired by continuous suture. There was no residual shunt at the patch repair site. After the operation, the patient was monitored closely in the ICU. The results of routine echocardiography were satisfactory. Antibiotic treatment was stopped when the blood culture result is negative. The general condition of the patient was stable, and the patient was cured.

## Discussion

Choosing the operative plan and its timing for pregnancy-associated IE continues to be a difficult problem to solve [1]. The operation plan needs to be discussed jointly by cardiologists, gynecologists, cardiologists, anesthesiologists and CPB doctors. Additionally, the requirements of patients and their families should be considered. The choice of operation time should take into account the severity of the basic heart disease, cardiac function, gestational age, complications and the hemodynamic status of the pregnant woman [2]. During the third trimester of pregnancy, CPB [3, 4] easily causes uterine contraction and premature delivery. With improvements in neonatal nursing, the survival rate of newborns over 28 weeks has greatly improved. Therefore, for pregnant women with IE, the fetal heart rate and uterine contractions should be closely monitored, and hemodynamic stability should be maintained through diuretics and antibiotics. These patients can then undergo a cesarean section and be allowed to recover before undergoing cardiac surgery, to reduce the risk of uncontrollable uterine bleeding that could occur if cesarean section and cardiac surgery with CPB were performed close together [5]. However, if the hemodynamics are still unstable after drug treatment, the cesarean section should be followed by systemic heparinization for CPB cardiac surgery. Although the risk of this operation is high, it can reduce the risk to pregnant women and their fetuses compared with conservative medical treatment. During the operation, measures such as maintaining a normal temperature or light hypothermia, maintaining a high flow rate, maintaining high perfusion pressure, avoiding overdilution of blood, routinely using progesterone, preventing uterine contractions, monitoring the whole fetal heart rate, and strengthening perioperative management can be taken to reduce the risk associated with the operation. Echocardiography has high sensitivity and specificity for the detection of vegetation. These findings can provide important reference information for the clear diagnosis and guidance of cardiac surgery, and echocardiography is an indispensable auxiliary examination for the diagnosis and treatment of pregnancy complicated by IE.

## Conclusion

We have successfully completed surgery on a rare cases of IE during pregnancy, in which echocardiography played an important role in the monitoring of the condition. This case shows that multidisciplinary collaboration, accurate assessment of the condition, careful perinatal monitoring and management, standardized anti-infection treatment, and the selection of an appropriate time for delivery and heart surgery are conducive to reducing maternal and infant mortality.

## Data Availability

The datasets during and/or analyzed during the current study are available from the corresponding author upon reasonable request.

## Abbreviations

IE: Infective endocarditis
PDA: Patent ductus arteriosus
MVP: Mitral valvuloplasty
CPB: Cardiopulmonary bypass
